# Supplementary material for: Impacts of air pollutants from rural Chinese households under the rapid residential energy transition
Source: Nat Commun. 2019 Jul 30;10:3405. doi: 10.1038/s41467-019-11453-w (PMC6667435; doi:10.1038/s41467-019-11453-w)
Supplement: Supplementary file 1 — Supporting Information [file 41467_2019_11453_MOESM1_ESM.pdf]

Supporting information for

Impacts of Air Pollutants from Rural Chinese Households under the Rapid Residential Energy Transition

Shen *et al.*,

The material includes 10 figures, 4 tables, and 1 supplementary note.

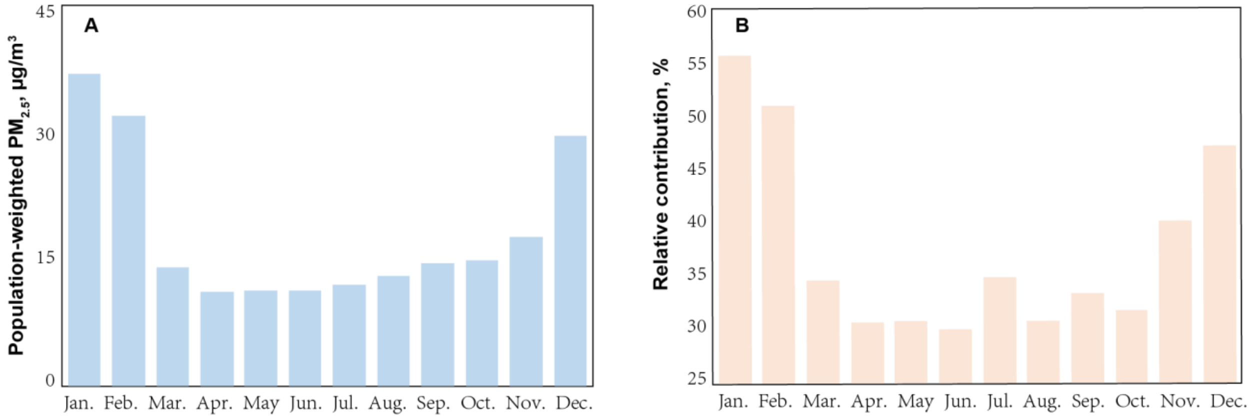

Supplementary Figure 1 Monthly variation of rural residential emission contributions to the total population-weighted $PM_{2.5}$ in mainland China in 1992. Results are shown for the monthly average absolute (**A**) and relative contributions (**B**). Source data are provided as a Source Data file.

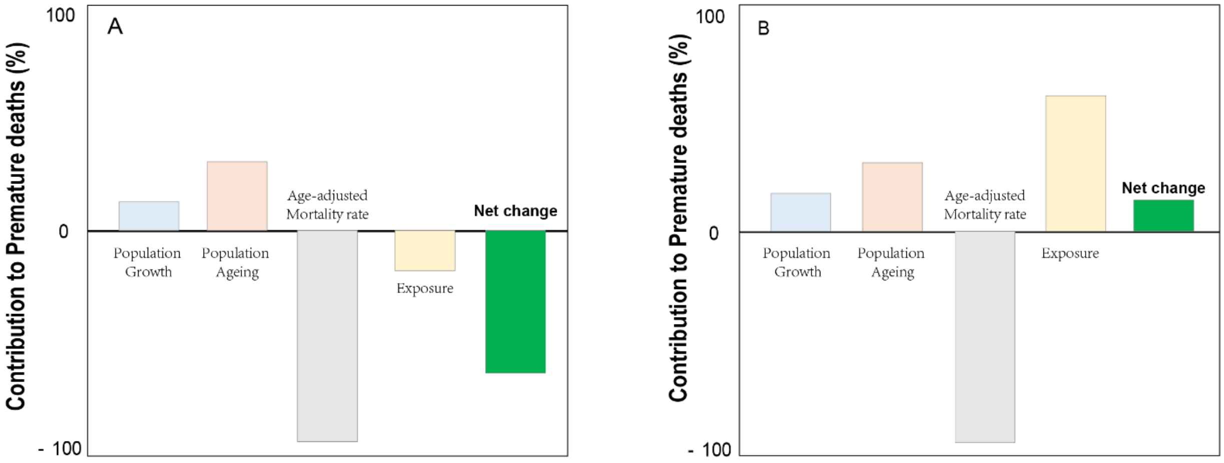

Supplementary Figure 2 Contributions of population growth, population ageing, age-adjusted mortality rate, and exposure to the net change in premature deaths from 1992 to 2012. The exposure was due to (A) residential emission, and (B) all sources. Source data are provided as a Source Data file.

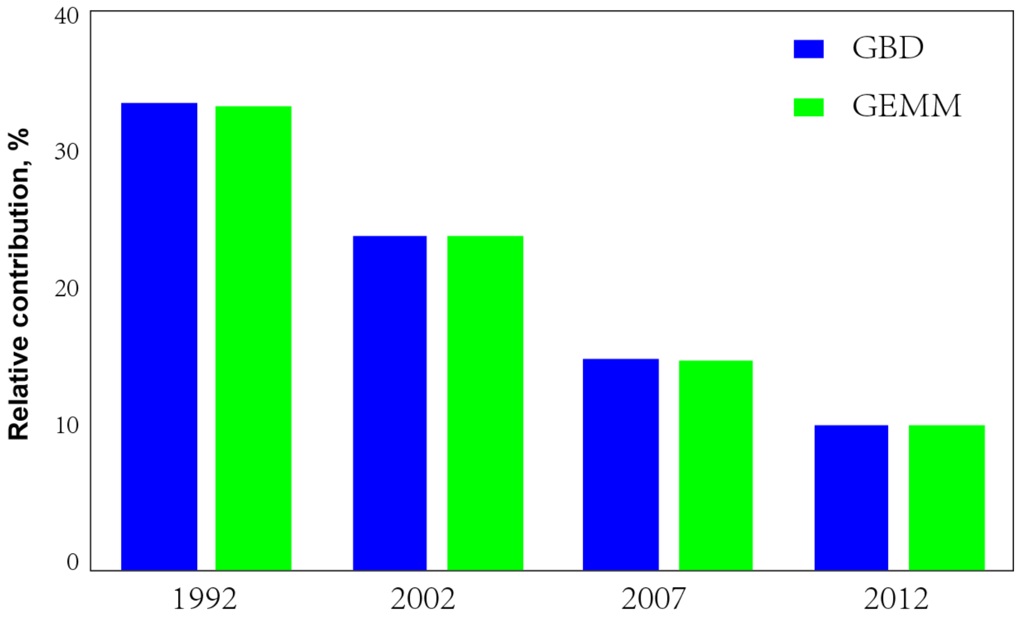

Supplementary Figure 3 Relative contributions of rural residential emissions to the total premature deaths induced by exposure to ambient air $PM_{2.5}$ in China from 1992 to 2012. The estimation was carried out following the Global Exposure Mortality Model (GEMM) and Global Burden of Disease (GBD) models, respectively. Source data are provided as a Source Data file.

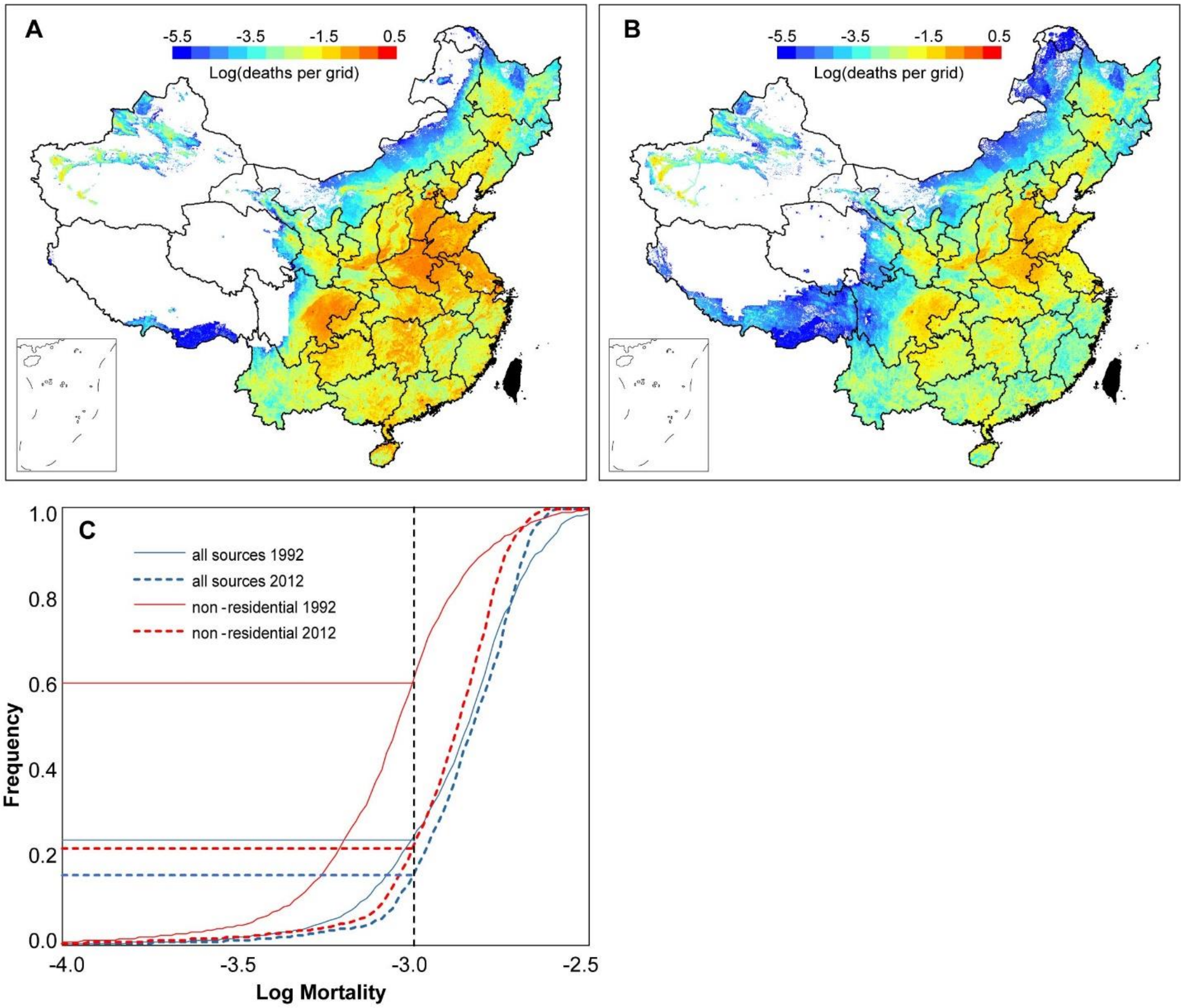

Supplementary Figure 4 Premature death associated with exposure to $PM_{2.5}$ from rural residential emissions in China. Spatial distributions of log-scale premature deaths induced by ambient $PM_{2.5}$ originating from rural residential emissions are compared between 1992 (**A**) and 2012 (**B**). The five $PM_{2.5}$ exposure-associated diseases include acute lower respiratory infections (ALRIs), ischemic heart disease (IHD), cerebrovascular disease (Stroke), chronic obstructive pulmonary disease (COPD), and lung cancer (LC). The cumulative frequency distributions of the grid mortality induced by $PM_{2.5}$ from all sources (blue lines) and all but the rural residential source (red lines) for 1992 (solid lines) and 2012 (dashed lines) and the cumulative frequency distributions of mortality rate in 1992 and 2012 are also shown (**C**). The differences between the solid and dashed lines had shrunk from 1992 (solid) to 2012 (dashed), indicating reduced relative contributions of rural residential sources. Source data are provided as a Source Data file.

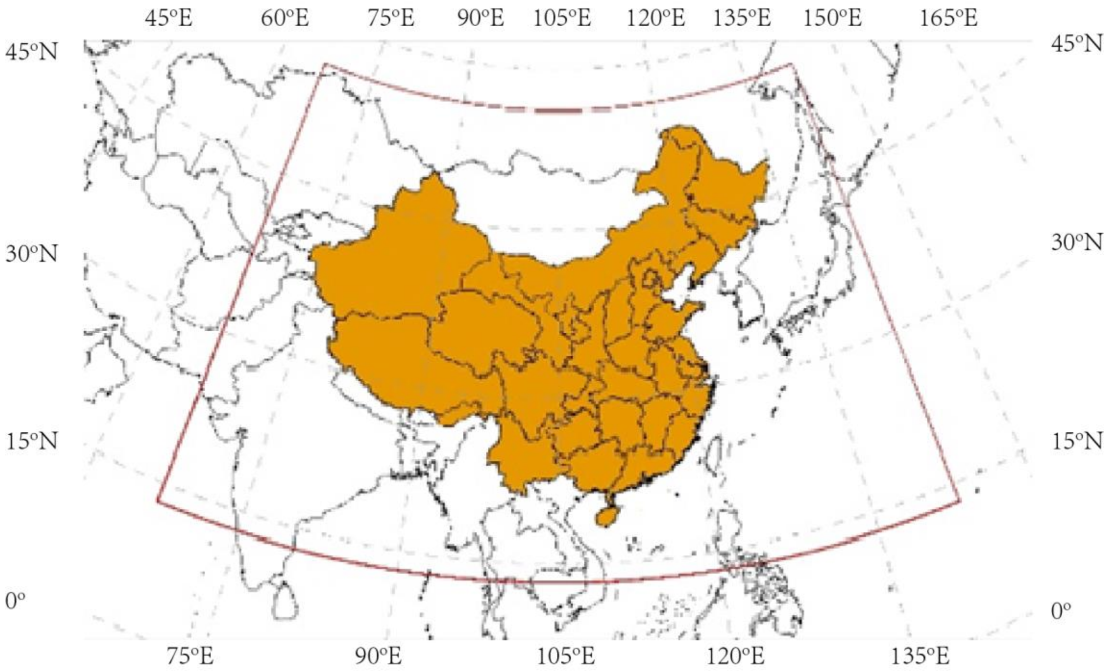

Supplementary Figure 5 Area modelled in this study covering a region between 13°N and 56°N and 67°E and 143°E.

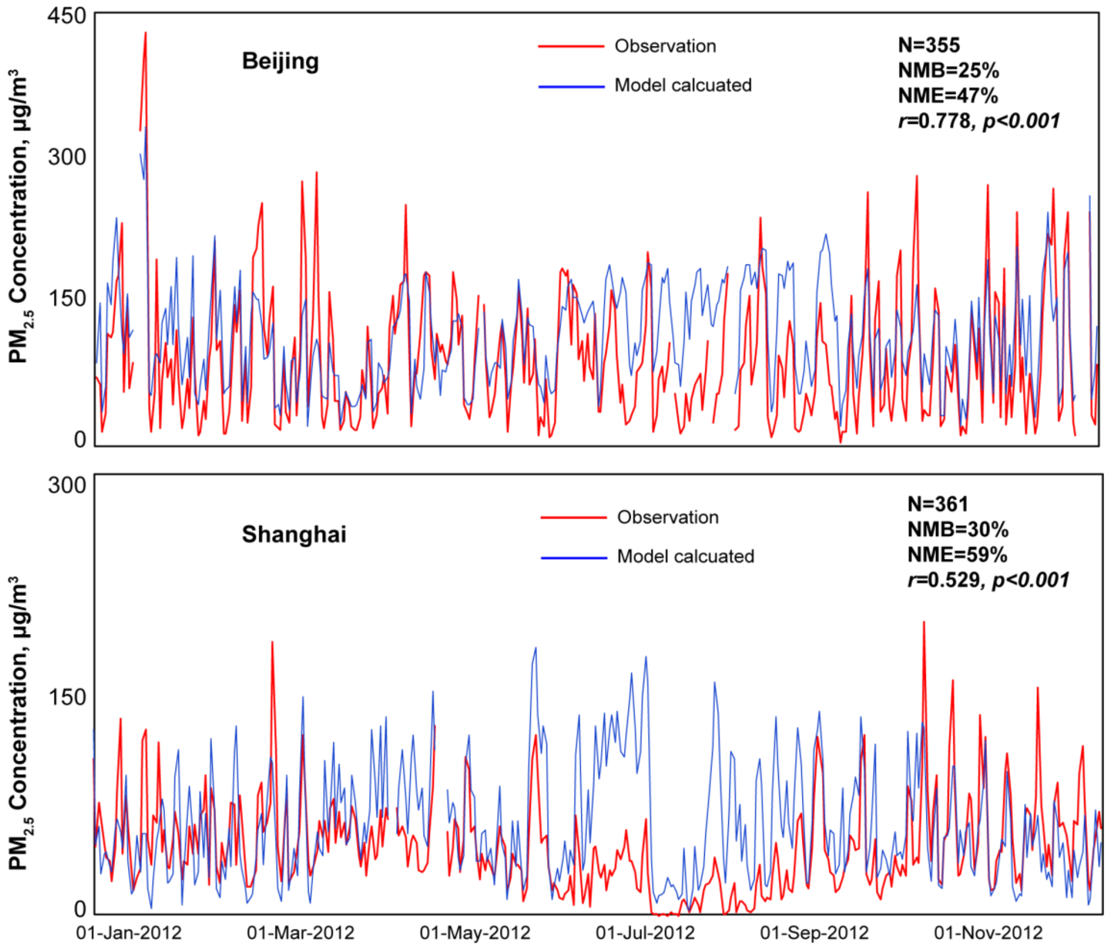

Supplementary Figure 6 Comparison between the model-calculated and observed time series of daily mean ambient $PM_{2.5}$ concentrations in Beijing and Shanghai in 2012. In 2012, these were the only available time-series data in mainland China. Sample size (N), normalized mean bias (NMB) and normalized mean error (NME) are calculated. Correlation coefficients are Pearson coefficients using log-transformed concentrations. Source data are provided as a Source Data file.

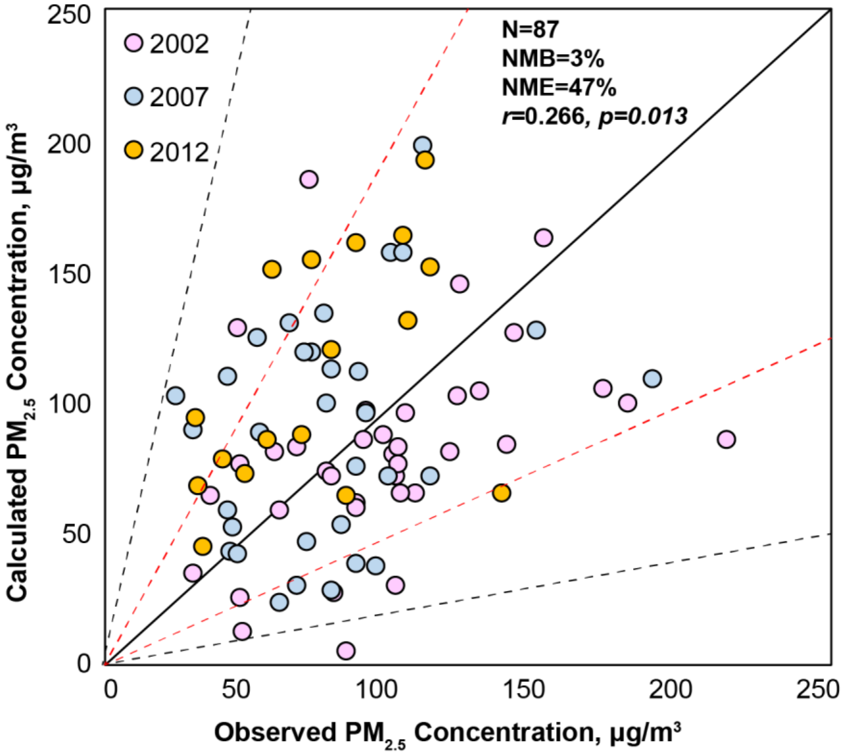

Supplementary Figure 7 Comparison between the model-calculated $PM_{2.5}$ concentrations and those from field measurements reported in the literature. The dashed red and black lines represent the error ranges of two and five times, respectively. Sample size (N), normalized mean bias (NMB) and normalized mean error (NME) are calculated. Correlation coefficients are Pearson coefficients using log-transformed concentrations. Source data are provided as a Source Data file.

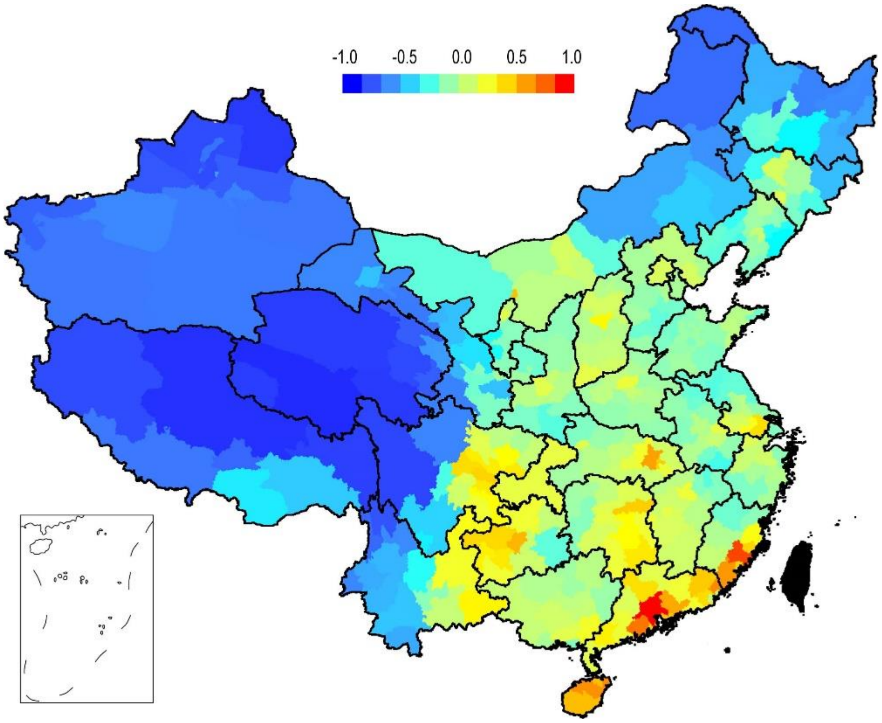

Supplementary Figure 8 Relative difference between the model-calculated $PM_{2.5}$ concentrations and satellite retrieved $PM_{2.5}$ in 2012. The difference is calculated as modelled annual average minus satellite retrieved concentrations. Retrieved concentrations are from Ma et al., 2016.[1] Source data are provided in a Source Data file.

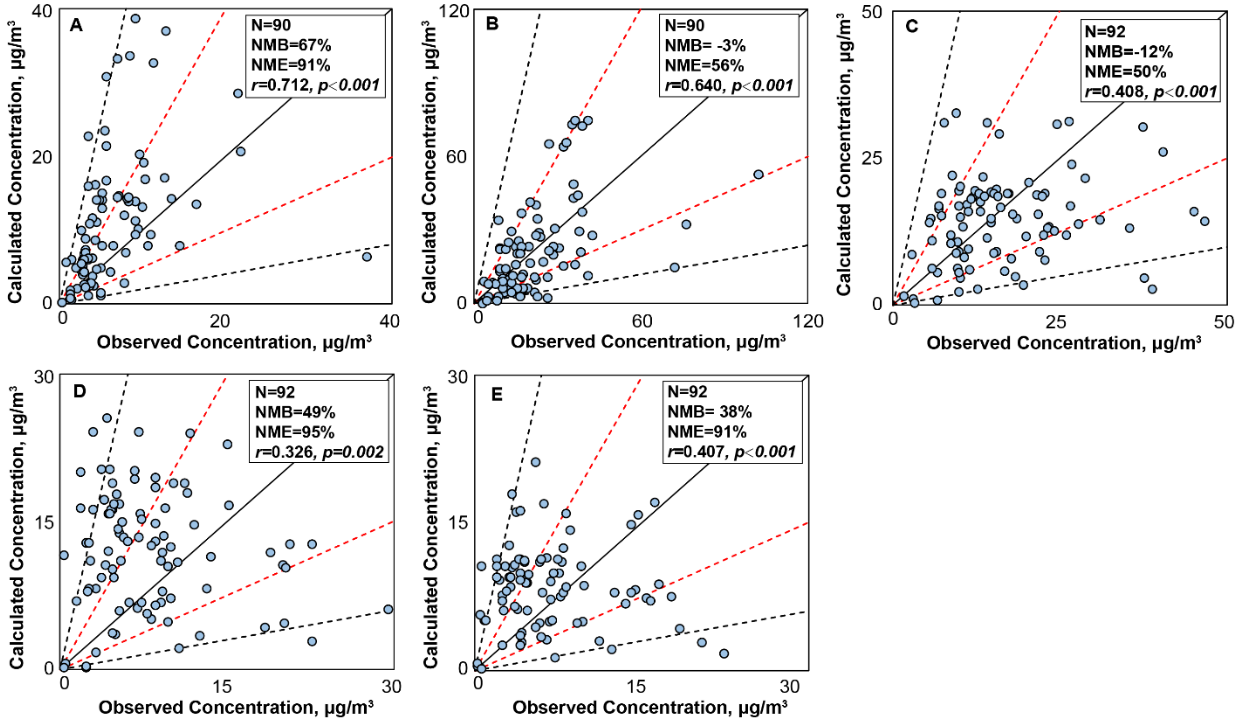

Supplementary Figure 9 Comparison of the modelled concentrations of major PM2.5 components including black carbon (**A**), organic carbon (**B**), sulfate (**C**), nitrate (**D**), and ammonium (**E**) with those observed. The dashed red and black lines represent the error ranges of two and five times, respectively. Sample size (N), normalized mean bias (NMB) and normalized mean error (NME) are calculated. Correlation coefficients are Pearson coefficients using log-transformed concentrations. Source data are provided as a Source Data file.

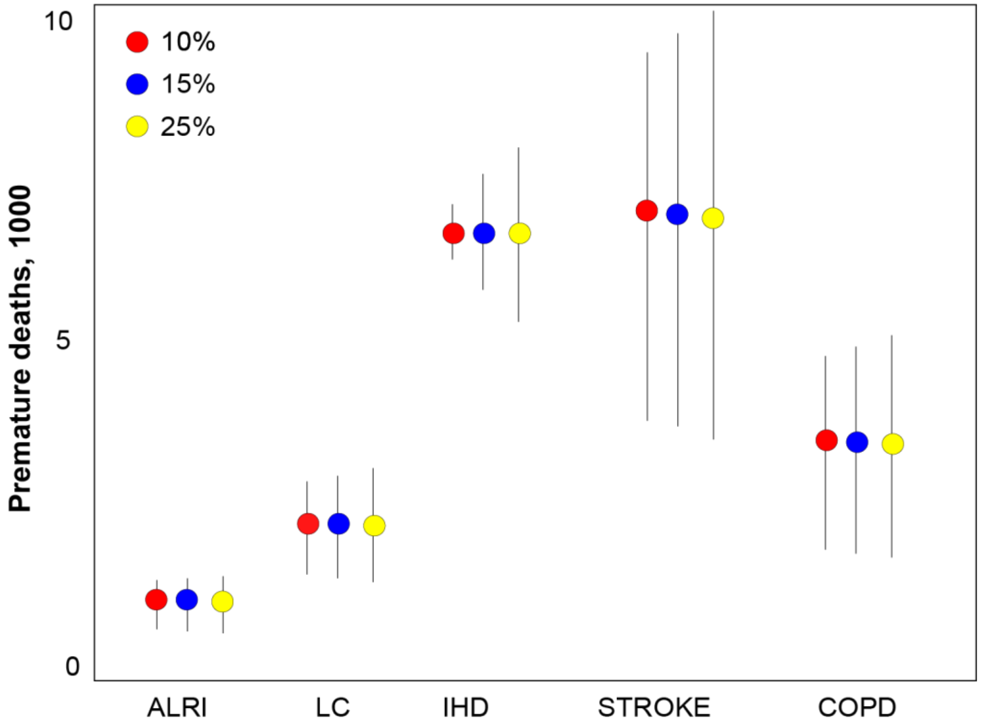

Supplementary Figure 10 Estimated premature deaths attributable to rural residential emissions in 2012. Results shown are the medium number with 95% uncertainty interval. The uncertainty intervals were derived from uncertainties in parameters in the dose-exposure relationship as well as the $PM_{2.5}$ exposure level in which 10%, 15%, and 25% variations in $PM_{2.5}$ concentration in individual grid cells were assumed and compared. The premature deaths estimated here are summarized from five diseases iincluding chronic obstructive pulmonary disease (COPD), cerebrovascular disease (stroke), ischaemic heart disease (IHD), lung cancer (LC), and acute lower respiratory infections (ALRIs). Source data are provided as a Source Data file.

Supplementary Table 1 Contributions of the rural residential sector to population-weighted $PM_{2.5}$ in individual provinces of mainland China in 2012. The results are presented in both absolute and relative terms for annual means and monthly means in heating (January as an example) and non-heating (October as an example) periods.

		Annual, 2012		January, 2012		October, 2012	
		μg/m³	%	μg/m³	%	μg/m³	%
**Northeast**	Heilongjiang	10±7.2	27±13%	15±23	38±23%	6.2±8.3	15±16%
	Jilin	11±2.9	26±11%	18±10	34±15%	5.0±3.8	14±14%
	Liaoning	16±15	24±15%	14±8.3	27±19%	5.9±6.6	13±15%
	Inner Mongolia	19±30	31±25%	45±75	51±38%	13±30	21±30%
**North**	Beijing	4.7±3.9	5.2±5.3%	8.9±8.6	10±11%	1.9±3.4	3.0±5.5%
	Tianjin	8.5±4.5	8.7±6.4%	14±12	14±13%	3.5±4.9	5.1±7.1%
	Hebei	12±6.1	14±6.7%	30±17	27±13%	7.3±5.6	10±7.2%
	Shandong	17±7.1	20±9.6%	31±13	30±12%	7.7±7.2	11±11%
	Shanxi	14±14	18±16%	35±39	35±27%	10±12	17±16%
**Northwest**	Shaanxi	9.2±5.9	15±7.8%	25±14	34±17%	6.7±5.9	11±7.9%
	Gansu	7.0±4.6	24±12%	18±12	50±23%	4.8±3.2	15±9.4%
	Ningxia	3.7±2.6	14±10%	8.6±6.6	31±25%	2.4±2.0	9.3±7.3%
	Qinghai	3.3±2.6	23±14%	8.6±6.9	46±23%	3.1±2.5	22±16%
	Xingjiang	2.9±2.8	34±25%	6.9±7.9	45±36%	1.7±1.9	20±18%
**East**	Shanghai	17±2.5	23±3.9%	3.6±2.1	7.1±6.5%	10±4.2	14±6.7%
	Jiangsu	15±2.1	19±5.3%	11±7.8	16±11%	5.1±3.5	6.8±5.8%
	Zhejiang	16±5.9	24±11%	4.6±4.2	9.4±9.0%	9.0±7.7	15±14%
	Anhui	14±6.9	17±7.3%	22±17	28±17%	11±8.8	13±9.0%
	Jiangxi	8.6±7.2	14±6.8%	10±10	16±7.9%	4.6±9.4	5.4±5.1%
**Middle**	Henan	14±5.0	15±5.9%	36±12	33±12%	12±6.2	13±7.1%
	Hubei	9.1±11	10±6.8%	22±26	25±15%	9.3±16	9.1±9.3%
	Hunan	8.0±2.5	10±4.2%	17±7.5	22±11%	7.2±5.1	7.7±5.2%
**South**	Fujian	23±8.4	44±9.2%	4.2±3.8	10±8.9%	18±8.4	30±14%
	Guangdong	27±13	35±16%	13±20	16±19%	29±25	26±20%
	Guangxi	12±3.2	21±6.5%	5.1±4.6	7.9±6.8%	20±7.4	23±7.7%
	Hainan	23±15	49±6.1%	29±16	39±13%	33±14	51±9.1%
**Southwest**	Sichuan	10±5.8	14±7.9%	26±16	30±14%	10±6.8	11±7.6%
	Chongqin	6.2±3.8	7.5±4.4%	16±9.8	19±12%	6.1±5.1	5.5±3.7%
	Guizhou	11±7.1	15±6.0%	15±27	16±16%	12±7.1	15±8.8%
	Yunnan	11±6.3	42±13%	12±9.1	52±19%	13±7.6	41±18%
	Xizang	2.5±2.3	53±3.0%	2.6±2.6	52±4.3%	3.1±3.4	55±5.3%
**National average**		14±10	21±14%	19±21	26±20%	11±13	21±14%

Supplementary Table 2. Evaluation of the daily mean surface pressure, temperature, relative humidity and average wind speed for the four study years between the WRF meteorological inputs and observations from National Meteorological Information Center.[2]

	Pressure			Temperature			Relative Humidity			Wind Speed		
	N	NMB	NME	N	NMB	NME	N	NMB	NME	N	NMB	NME
1992	60389	-3%	3%	60390	-9%	21%	60390	-3%	16%	57168	39%	56%
2002	60225	-3%	3%	60223	-11%	20%	60224	-2%	15%	57259	43%	59%
2007	60590	-3%	3%	60590	-7%	17%	60590	-9%	18%	60024	32%	48%
2012	60747	-3%	3%	60747	-9%	19%	60738	-7%	16%	60720	32%	48%

Supplementary Table 3. Evaluation of the modelled ambient $PM_{2.5}$ against the estimated historical $PM_{2.5}$ in past studies. Literature results are from those based on satellite-based Aerosol Optical Depth (AOD) using a chemical transport model (CTM), from AOD with a statistical model, and that retrieved from the historical visibility records.

	Modelled $PM_{2.5}$ (this study) ***vs.*** AOD-$PM_{2.5}$ (CTM, van Donkelaar et al. 2015[3])			Modelled $PM_{2.5}$ (this study) ***vs.*** AOD-$PM_{2.5}$ (statistical model, Ma et al., 2016[1])			Modelled $PM_{2.5}$ (this study) ***vs.*** visibility-based $PM_{2.5}$ (Liu et al., 2017[4])		
	N	NMB	NME	N	NMB	NME	N	NMB	NME
1992	/	/	/	/	/	/	614	-45%	56%
2002	94294	-43%	50%	/	/	/	614	-51%	57%
2007	94294	-44%	51%	87628	-61%	63%	396	-32%	53%
2012	94294	0%	65%	84789	-35%	46%	378	-5%	46%

Supplementary Table 4. Estimated premature deaths (medium with 95% uncertainty interval) attributable to rural residential emissions for the four study years following the Global Exposure Mortality Model (GEMM) and Global Burden of Disease (GBD) models. The results are shown for chronic obstructive pulmonary disease (COPD), cerebrovascular disease (stroke), ischaemic heart disease (IHD), lung cancer (LC), and acute lower respiratory infections (ALRIs).

Year		GEMM	GBD
1992	ALRI	77,000 (46,000-98,000)	40,000 (30,000-50,000)
	LC	34,000 (22,000-43,000)	20,000 (10,000-30,000)
	IHD	150,000 (140,000-160,000)	80,000 (30,000-120,000)
	STROKE	200,000 (110,000-260,000)	110,000 (60,000-180,000)
	COPD	110,000 (60,000-150,000)	90,000 (50,000-130,000)
	**TOTAL**	**570,000 (370,000-710,000)**	**340,000 (200,000-500,000)**
2002	ALRI	33,000 (20,000-42,000)	20,000 (10,000-30,000)
	LC	36,000 (24,000-45,000)	20,000 (10,000-30,000)
	IHD	130,000 (120,000-140,000)	70,000 (40,000-100,000)
	STROKE	160,000 (90,000-220,000)	90,000 (50,000-140,000)
	COPD	82,000 (44,000-110,000)	70,000 (40,000-100,000)
	**TOTAL**	**440,000 (300,000-560,000)**	**270,000 (150,000-390,000)**
2007	ALRI	17,000 (10,000-21,000)	9,000 (7,000-20,000)
	LC	30,000 (20,000-38,000)	20,000 (10,000-30,000)
	IHD	94,000 (89,000-100,000)	50,000 (20,000-70,000)
	STROKE	110,000 (58,000-140,000)	60,000 (30,000-90,000)
	COPD	50,000 (27,000-67000)	40,000 (30,000-60,000)
	**TOTAL**	**300,000 (200,000-360,000)**	**170,000 (100,000-250,000)**
2012	ALRI	12,000 (7,000-15,000)	7,000 (5,000-9,000)
	LC	24,000 (16,000-30,000)	15,000 (10,000-20,000)
	IHD	67,000 (62,000-70,000)	30,000 (20,000-50,000)
	STROKE	70,000 (39,000-93,000)	40,000 (20,000-50,000)
	COPD	36,000 (19,000-48,000)	30,000 (20,000-40,000)
	**TOTAL**	**210,000 (140,000-260,000)**	**120,000 (70,000-180,000)**

Supplementary Note 1:

Normalized marginal method

Due to the nonlinear relationship between the emissions and the modelled $PM_{2.5}$ concentrations, a normalized marginal method was applied to calculate the relative contributions of rural residential sources to ambient $PM_{2.5}$ concentrations. We performed the simulations using three emission scenarios of: a 20% reduction in the rural residential sources (r-20%); a 20% reduction in all but the residential sources (o-20%); and all sources.

We assessed the relative contributions of rural residential sources for each simulated grid using the following equation: $\boldsymbol{RC}r=(\boldsymbol{C}_{all}-\boldsymbol{C}_{r-20\%})/(2\times\boldsymbol{C}_{all}-\boldsymbol{C}_{r-20\%}-\boldsymbol{C}_{o-20\%})$, where $\boldsymbol{RC}_r$ is the relative contribution of rural residential emissions to ambient $PM_{2.5}$; C_{all}, $C_{r-20\%}$ and $C_{o-20\%}$ are the ambient $PM_{2.5}$ concentrations in the three scenarios of all sources, a 20% reduction in the rural residential sources, and a 20% reduction in all but the residential sources, respectively. The absolute contribution of rural residential emissions was calculated as $\boldsymbol{RC}_r\times\boldsymbol{C}_{all}$ for each grid.

The OSCAR model was run to assess the contribution of rural residential emissions to radiative forcing. The model was run three times with the three scenarios, the same as that in the chemical transport modelling, and the relative contribution was calculated as $(\boldsymbol{RF}_{all}-\boldsymbol{RF}_{r-20\%})/(2\times\boldsymbol{RF}_{all}-\boldsymbol{RF}_{r-20\%}-\boldsymbol{RF}_{o-20\%})$, where $\boldsymbol{RF}_{all}$, $\boldsymbol{RF}_{r-20\%}$ and $\boldsymbol{RF}_{o-20\%}$ are the radiative forcing values in the three scenarios of all sources, a 20% reduction in the rural residential sources, and a 20% reduction in all but the residential sources, respectively.

Supplementary Reference

1. Ma, Z., Liu, R., Liu, Y., & Bi, J. Effects of air pollution control policies on $PM_{2.5}$ pollution improvement in China from 2005 to 2017: a satellite-based perspective. Atmos. Chem. Phys. ***19***, 6861-6877, (2019).
2. National Meteorological Information Center, China Earth International Exchange Station Climate Data Daily Value Data Set (V3.0), http://data.cma.cn/. Accessed Jun. 2019.
3. van Donkelaar, A. et al. Global fine particulate matter concentrations from satellite for long-term exposure assessment. Environ. Health Perspect. ***123***, 135-143, (2015).
4. Liu, M., Bi. J., Ma, Z. Visibility-based PM2.5 concentrations in China: 1957-1964 and 1973-2014. Environ. Sci. Technol. ***51***, 13161-13169, (2017).
